# Hydrocarbon ingestions among individuals younger than 20 years old reported to United States Poison Centers, 2000–2021

**DOI:** 10.1186/s40621-023-00461-6

**Published:** 2023-10-12

**Authors:** Samiza B. Palmer, Henry A. Spiller, Sandhya Kistamgari, Marcel J. Casavant, Natalie I. Rine, Jingzhen Yang, Motao Zhu, Gary A. Smith

**Affiliations:** 1 Center for Injury Research and Policy, The Abigail Wexner Research Institute at Nationwide Children’s Hospital, 700 Children’s Drive, Columbus, OH 43205 USA; 2 Drexel University College of Medicine, Philadelphia, PA USA; 3 Department of Pediatrics, The Ohio State University College of Medicine, Columbus, OH USA; 4 Central Ohio Poison Center, Nationwide Children’s Hospital, Columbus, OH USA; 5 Child Injury Prevention Alliance, Columbus, OH USA

**Keywords:** Hydrocarbons, Ingestion, Aspiration, Poisoning, Pediatric

## Abstract

**Background:**

Hydrocarbon-based products have many household and commercial uses and exposure to these substances is common. Severe clinical effects can occur if these products are ingested. This study investigated the characteristics and trends of hydrocarbon ingestions reported to United States Poison Centers.

**Methods:**

Data from the National Poison Data System were analyzed for cases of hydrocarbon ingestion among individuals < 20 years old reported to United States Poison Centers from January 1, 2000 through December 31, 2021.

**Results:**

There were 284,085 hydrocarbon ingestions reported during the 22-year study period in which a hydrocarbon was the first-ranked substance. Most of these cases occurred among children < 6 years old (83.2%), males (64.6%), at a residence (96.5%), were single-substance exposures (98.3%), and were managed on-site rather than in a health care facility (74.9%). However, 4.5% of cases were associated with a serious medical outcome, including 34 deaths. Thirty-two deaths were among children < 6 years old and most were associated with aspiration. Gasolines accounted for 24.6% of total cases, followed by lubricating oils and/or motor oils (19.9%), other types of hydrocarbons (14.9%), lamp oils (11.3%), and lighter fluids and/or naphtha (10.3%). The rate of hydrocarbon ingestions among United States youth < 20 years old decreased significantly (*p* < 0.0001) by 66.5% from 2000 to 2021. The greatest rate decrease was observed among lamp oils (− 78.4%, *p* < 0.0001), followed by gasolines (− 75.9%, *p* < 0.0001).

**Conclusions:**

Although the rate of hydrocarbon ingestions decreased during the study period and most reported cases resulted in non-serious outcomes, the number of cases remains high with a non-trivial minority (4.5%) of cases associated with a serious medical outcome, including death. Most deaths were among children < 6 years old. This underscores the need to increase primary prevention efforts, especially for young children.

## Background

Hydrocarbon-based products contain petroleum distillates and have many household and commercial uses and exposure to these substances is common. Severe clinical effects can occur if these products are ingested (Forrester 2022; Jolliff et al. 2013; Makrygianni et al. 2016; Reddy et al. 2020; Schwebel and Swart 2009; Sheikh et al. 2013; Slima et al. 2021; Tenenbaum et al. 2021). The most devastating effects occur when the ingested hydrocarbon is aspirated into the lungs. The resulting pneumonitis can have fatal consequences (Ghezzi et al. 2019; Perrot and Palmer 1992). In 2021, hydrocarbons were among the top 10 most common substance categories associated with death among children < 6 years old reported to United States Poison Centers (PCs) (Gummin et al. 2021). Due to their low viscosity and many colors and fragrances, these liquids can be mistaken for juices and other drinks (Sheridan et al. 2021). This risk is heightened among young children, who have newly acquired mobility, are curious, and do not recognize danger (Tenenbaum et al. 2021). Unintentional exposures to hydrocarbons associated with exploratory behaviors peak at ages 1–2 years (Jolliff et al. 2013). Although emergency department visits and calls to PCs associated with hydrocarbon ingestion among children < 6 years old decreased from 2000 to 2009 in the US, one study reported a tenfold increase in emergency department visits associated with lamp oil ingestion in this age group from 2014 to 2017 (Forrester 2022). Therefore, previously published research suggests that the decrease in hydrocarbon ingestion during the past two decades may not have been uniform among all substances in this category and that these exposures remain an important source of poisoning, especially among young children.

The most recent national study of US pediatric hydrocarbon ingestions evaluated data only through 2009 (Jolliff et al. 2013). More recent studies have focused more narrowly on specific hydrocarbons such as lamp oil (Forrester 2022) or kerosene (Reddy et al. 2020; Slima et al. 2021; Tenenbaum et al. 2021), exposures associated with a natural disaster (Cox et al. 2008); case reports (Ghezzi et al. 2019; Murthy et al. 2018); or exposures managed by a single PC/hospital (Tenenbaum et al. 2021) or in other countries, such as India (Reddy et al. 2020; Jayashree and Singhi 2011), Israel (Tenenbaum et al. 2021), or Egypt (Slima et al. 2021). The objective of this study is to update and expand our understanding of the characteristics and trends of hydrocarbon ingestions in the US among individuals < 20 years old by analyzing national data for the years 2000 through 2021.

## Methods

### Data source

Data were obtained from the National Poison Data System (NPDS), which is maintained by America’s Poison Centers (formerly known as the American Association of Poison Control Centers). This national database collects standardized information from telephone calls to PCs across the US and its territories (America’s Poison Centers 2023). US Census data were also used to calculate age group-specific and sex-specific population-based rates. This study was deemed exempt from approval by the institutional review board of the Abigail Wexner Research Institute at Nationwide Children’s Hospital.

### Case selection criteria

Cases involving exposures to hydrocarbons among individuals younger than 20 years old reported to US PCs from January 1, 2000 through December 31, 2021 (*n* = 425,948) were obtained from the NPDS. Hydrocarbons in the following NPDS generic substance categories were included: diesel fuels, gasolines, kerosenes, lamp oils, lighter fluids and/or naphtha, lubricating oils, mineral seal oil, mineral spirits, other types of halogenated hydrocarbons, other types of hydrocarbons, turpentine, and unknown types of hydrocarbon. Among the cases provided by the NPDS, 297,640 met study inclusion criteria. Cases (referred to as ingestion cases in this article) were included if at least one route of exposure was (1) ingestion or (2) aspiration (with ingestion), which resulted in the exclusion of 120,492 cases associated with other routes of exposure. The following cases were also excluded from the study: (1) exposure occurred outside the 50 US states or District of Columbia (*n* = 1451), or (2) medical outcome was coded as “confirmed non-exposure” (*n* = 355) or “unrelated effect” (*n* = 5044), reason for exposure coded as “adverse reaction—food” (*n* = 207) or “adverse reaction—drug” (*n* = 92) or “unintentional—bite/sting” (*n* = 11) or “unintentional—therapeutic error” (*n* = 484) or “unintentional—food poisoning” (*n* = 172). The NPDS defines “confirmed non-exposure” as an exposure in which “there is reliable and objective evidence that the exposure never occurred and that any symptoms exhibited by the patient were not related to the reported exposure,” and “unrelated effect” as a report for which “based upon all the information available, the exposure was probably not responsible for the effect(s)” (American Association of Poison Control Centers 2014).

### Study variables

Age groups were categorized as: (1) < 6 years, (2) 6–12 years, and (3) 13–19 years. The reason for exposure was grouped as (1) unintentional-general (includes exploratory behaviors), (2) unintentional-other (includes environmental/occupational/misuse), (3) unintentional-unknown, (4) intentional-suspected suicide, (5) intentional-misuse, (6) intentional-abuse, (7) intentional-unknown, (8) other, and (9) unknown. The NPDS defines intentional-misuse as “an exposure resulting from the intentional improper or incorrect use of a substance for reasons other than the pursuit of a psychotropic effect,” and intentional-abuse as “an exposure resulting from the intentional improper or incorrect use of a substance where the patient was likely attempting to gain a high, euphoric effect or some other psychotropic effect, including recreational use of a substance for any effect.” () Exposure sites were grouped into (1) residence (includes other or own residence), (2) school, (3) other (includes health care facility, public area, restaurant, workplace, and other), and (4) unknown.

The level of health care received was based on NPDS-defined categories and was grouped into (1) no health care facility (HCF) treatment received, (2) treated/evaluated at a HCF and released, (3) admitted (includes admitted to critical care unit [CCU], non-CCU, and psychiatric care facility), (4) refused referral/did not arrive at a HCF, and (5) unknown (includes left against medical advice and lost to follow-up and unknown) (American Association of Poison Control Centers 2014). The “no HCF treatment received” category was calculated as the sum of the “managed on-site (not in a HCF)” and “other” categories of the NPDS management site variable. If the management site was “unknown,” then that case was included in the “lost to follow-up/ left against medical advice/ unknown” category of the level of health care received variable. We also grouped admissions as (1) admitted to a CCU or non-CCU (to capture medical-related admissions) and (2) admitted directly to a psychiatric facility (to capture psychiatric-related admissions).

Medical outcome was based on NPDS-defined categories and was grouped into: (1) no effect (no symptoms developed as a result of the exposure), (2) minor effect (minimally bothersome symptoms that generally resolve rapidly with no residual disability), (3) moderate effect (more pronounced, prolonged, or systemic than minor symptoms), (4) major effect (symptoms are life-threatening or result in significant disability or disfigurement), (5) death, (6) not followed (includes minimal clinical effects possible and judged as a non-toxic exposure), and (7) unknown (includes unable to follow [judged as a potentially toxic exposure]) (American Association of Poison Control Centers 2014). For analyses, moderate effect, major effect, and death were combined into the category “serious medical outcome.”

### Statistical analysis

NPDS data were analyzed using IBM Statistics SPSS 28 for Windows (IBM Corp, Armonk, NY) and SAS 9.4 (SAS Institute Inc, Cary, NC) statistical software. Population-based rates were calculated using US Census Bureau July 1 intercensal and postcensal population estimates from 2000 through 2021 (United States Census Bureau 2023a, b, c). Analyses of the general characteristics of hydrocarbon ingestions were restricted to those cases in which the first-ranked substance was a hydrocarbon. A first-ranked substance is defined by the NPDS as the substance that is most likely responsible for the observed clinical effects based on the judgement of a Certified Specialist in Poison Information at the PC. Cases in which a hydrocarbon is first-ranked include both (1) single-substance (hydrocarbon only) exposures and (2) multiple-substance exposures in which a hydrocarbon is the first-ranked substance. To avoid misattribution associated with substance-substance interactions, only single-substance exposures were included in analyses of related clinical effects. All hydrocarbon ingestion cases (including single-substance, first-ranked multiple-substance, and non-first-ranked multiple-substance) were included in analyses of trends over time. Trends were analyzed using simple or piecewise linear regression models, as appropriate. Break points for piecewise regression models were based on scatter plots of annual rates. Statistical significance was determined at α = 0.05. Other analyses included calculation of odds ratios (ORs) with 95% confidence intervals (CIs) to assess the magnitude of the relationship between risk factors and outcome measures, including sex and highest level of health care received, sex and medical outcome, age group and medical outcome, hydrocarbon category and medical outcome, hydrocarbon category and highest level of health care received, and hydrocarbon category and aspiration. A risk ratio (RR) with a 95% CI was calculated for the relationship between sex and reason for exposure because of the high prevalence and tendency for an OR to over-estimate the magnitude of this relationship.

## Results

### General characteristics of the study population

Among the 297,640 cases involving hydrocarbon ingestions among youth < 20 years old from 2000 through 2021 reported to US PCs, aspiration was associated with 8166 (2.7%) of these. A hydrocarbon was the first-ranked substance in 284,085 of the cases, averaging 12,913 cases per year (Table 1). Aspiration was involved in 8021 (2.8%) of the first-ranked hydrocarbon ingestion cases. Among the 284,085 first-ranked cases, most (83.2%) were < 6 years old, followed by 13–19 years old (10.7%). Ingestions peaked at ages one (34.8% of cases) and two (27.7% of cases) years (data not shown in table). Most first-ranked cases were single-substance exposures (98.3%, *n* = 279,393) and occurred most frequently among males (64.6%), at a residence (96.5%), and were managed on-site rather than in a HCF (74.9%) (Table 1). The most common related clinical effects associated with single-substance exposures were coughing/choking (32.3%), followed by vomiting (17.9%) and nausea (5.7%). Dyspnea (2.4%), drowsiness/lethargy (2.1%), and pneumonitis (1.1%) were also reported (data not shown in table).

**Table 1:** Characteristics of first-ranked hydrocarbon ingestions among individuals younger than 20 years old reported to the national poison data system by age group, 2000–2021

Characteristics	Age groups				
	< 6 years old	6–12 years old	13–19 years old	Unknown	Total
	*n* (%)^a^	*n* (%)^a^	*n* (%)^a^	*n*	*n* (%)^a^
*Route of exposure*					
Ingestion (without aspiration)	228,752 (96.9)	16,952 (98.8)	29,971 (98.4)	389	276,064 (97.2)
Aspiration (with ingestion)	7305 (3.1)	214 (1.2)	499 (1.6)	3	8021 (2.8)
*Sex*					
Male	146,412 (62.2)	11,877 (69.5)	24,494 (80.5)	297	183,080 (64.6)
Female	89,055 (37.8)	5202 (30.5)	5940 (19.5)	84	100,281 (35.4)
Unknown	590	87	36	11	724
*Type of exposure*					
Single-substance	232,756 (98.6)	16,854 (98.2)	29,405 (96.5)	378	279,393 (98.3)
Multiple-substance	3301 (1.4)	312 (1.8)	1065 (3.5)	14	4692 (1.7)
*Exposure site*					
Residence	231,647 (98.4)	15,227 (89.0)	25,910 (86.5)	266	273,050 (96.5)
School	287 (0.1)	840 (4.9)	1593 (5.2)	84	2804 (1.0)
Other^b^	3548 (1.5)	1036 (6.1)	2445 (8.3)	29	7058 (2.5)
Unknown	575	63	522	13	1173
*Reason for exposure*					
Unintentional	235,393 (99.7)	15,389 (90.1)	24,048 (79.4)	290	275,120 (96.9)
Unintentional—general	230,787 (97.8)	12,030 (70.4)	11,887 (39.2)	186	254,890 (89.8)
Unintentional—other^c^	4497 (1.9)	3311 (19.4)	12,053 (39.8)	101	19,962 (7.0)
Unintentional—unknown	109 (0.0)	48 (0.3)	108 (0.4)	3	268 (0.1)
Intentional	130 (0.0)	1140 (6.7)	5400 (17.8)	73	6743 (2.4)
Intentional—suspected suicide	15 (0.0)	80 (0.5)	1253 (4.1)	5	1353 (0.5)
Intentional—misuse	79 (0.0)	825 (4.8)	2980 (9.8)	40	3924 (1.4)
Intentional—abuse	9 (0.0)	136 (0.8)	949 (3.1)	25	1119 (0.4)
Intentional—unknown	27 (0.0)	99 (0.6)	218 (0.7)	3	347 (0.1)
Other	430 (0.2)	557 (3.2)	859 (2.8)	26	1872 (0.7)
Unknown	104	80	163	3	350
*Management site*					
Managed on-site (non-HCF)	175,633 (74.8)	13,375 (78.4)	22,256 (73.6)	289	211,553 (74.9)
Patient already in (enroute to) HCF when PC called	40,785 (17.3)	2499 (14.7)	4988 (16.5)	26	48,298 (17.1)
Patient was referred by PC to a HCF	17,117 (7.4)	852 (5.0)	2396 (7.9)	35	20,400 (7.2)
Other	1365 (0.6)	327 (1.9)	603 (2.0)	35	2330 (0.8)
Unknown	1157	113	227	7	1504
*Level of health care received*					
No HCF treatment received	176,998 (73.5)	13,702 (80.4)	22,859 (75.3)	324	213,883 (74.1)
Treated/evaluated and released	41,903 (17.4)	2661 (15.6)	4631 (15.3)	23	49,218 (17.1)
Admitted	10,188 (4.2)	273 (1.6)	1115 (3.7)	1	11,577 (4.0)
Admitted to a critical care unit	3785 (1.6)	70 (0.4)	279 (0.9)	0	4134 (1.4)
Admitted to a non-critical care unit	6385 (2.7)	170 (1.0)	318 (1.0)	0	6873 (2.4)
Admitted to a psychiatric facility	18 (0.0)	33 (0.2)	518 (1.7)	1	570 (0.2)
Patient refused referral/did not arrive at HCF	1566 (0.7)	130 (0.8)	630 (2.1)	14	2340 (0.8)
Unknown^d^	5402	400	1235	30	7067
*Medical outcome*					
No effect	84,764 (37.2)	4230 (25.5)	4644 (16.1)	61	93,699 (34.2)
Minor effect	43,638 (19.1)	4080 (24.6)	9375 (32.6)	63	57,156 (20.9)
Serious medical outcome	10,609 (4.7)	426 (2.6)	1358 (4.7)	6	12,399 (4.5)
Moderate effect	9636 (4.2)	409 (2.5)	1266 (4.4)	6	11,317 (4.1)
Major effect	941 (0.4)	17 (0.1)	90 (0.3)	0	1048 (0.4)
Death	32 (0.0)	0 (0.0)	2 (0.0)	0	34 (0.0)
Not followed (minimal clinical effects possible)	89,057 (39.0)	7878 (47.4)	13,393 (46.6)	221	110,549 (40.4)
Unknown^e^	7989	552	1700	41	10,282
Overall^f^	236,057 (83.2)	17,166 (6.1)	30,470 (10.7)	392	284,085 (100.0)

*HCF* health care facility, *PC* poison center

^a^Column percentages may not add to 100.0% due to rounding error

^b^Includes health care facility, public area, restaurant, workplace, and other

^c^Includes environmental/occupational/misuse

^d^Includes “left against medical advice” and “lost to follow-up” and “unknown”

^e^Unknown (includes unable to follow [potentially toxic exposure])

^f^Row percentages may not add to 100.0% due to rounding error

### Reason for exposure

Most (96.9%) exposures were unintentional, and the most common reason for exposure was unintentional-general (89.8%) (Table 1). The reason for exposure varied by age group. Unintentional-general exposures dominated among children < 6 years old (97.8%) and decreased with increasing age group, representing 39.2% of cases among teenagers 13–19 years old. In contrast, intentional reasons for exposure were more common among teenagers (17.8%) and decreased with decreasing age group. Among teenagers, misuse or abuse accounted for 12.9% of cases and suspected suicide or suicide attempts accounted for 4.1%. Children < 6 years old were more likely to be exposed due to an unintentional-general reason than older children (RR: 1.94; 95% CI 1.92–1.96), and teenagers 13–19 years old were more likely to be exposed due to an intentional reason than younger children (RR: 35.50; 95% CI 33.43–37.69).

### Highest level of health care received and medical outcome

Approximately three-fourths (74.1%) of hydrocarbon ingestions did not receive care at a HCF; however, 4.0% were admitted to a HCF (including 3.8% to a CCU or non-CCU and 0.2% directly to a psychiatric facility) (Table 1). Males (OR 1.05; 95% CI 1.03–1.07) were marginally more likely to receive care at a HCF than females, and children < 6 years old (OR 1.26; 95% CI 1.23–1.29) were more likely to receive care at a HCF than youth 6–19 years old. More specifically, males (OR 1.07; 95% CI 1.02–1.11) were slightly more likely to be admitted to a CCU or non-CCU than females, and children < 6 years old (OR 2.49; 95% CI 2.32–2.68) were more likely to be admitted to a CCU or non-CCU than youth 6–19 years old.

Compared with 6–19-year-olds, children < 6 years old were more likely to be exposed via aspiration (with ingestion) (OR 2.10; 95% CI 1.95–2.27) than ingestion without aspiration. Children who experienced aspiration (with ingestion) were more likely to have a serious medical outcome (OR 16.41; 95% CI 15.59–17.27), including death (OR 99.64; 95% CI 46.50–213.53), or be admitted to a CCU or non-CCU (OR 16.50; 95% CI 15.63–17.35) than children who ingested a hydrocarbon without aspiration.

Most hydrocarbon ingestions were associated with no effect (34.2%) or minor effect (20.9%) (Table 1). The proportion of cases with no effect decreased with increasing age group, ranging from 37.2% among children < 6 years old to 16.1% among 13–19-year-olds. In contrast, the proportion of cases with a minor effect increased with increasing age group, ranging from 19.1% among children < 6 years old to 32.6% among 13–19-year-olds. A serious medical outcome occurred in 4.5% of cases, including 34 deaths. Thirty-two of these deaths were reported among children < 6 years old and 2 were reported among teenagers 13–19 years old (Table 1). Fatality peaked at ages one (*n* = 15, 44.1% of deaths) and two (*n* = 7, 20.6% of deaths) years. Thirty-two deaths involved a single substance, and 25 deaths were associated with aspiration (with ingestion). Twenty-three of the 25 aspiration-related deaths were among children < 6 years old. Lamp oils was the leading hydrocarbon category associated with ingestion-related fatality, accounting for 12 deaths (35.3%), followed by gasolines (*n* = 5, 14.7%), mineral spirits (*n* = 5, 14.7%), kerosenes (*n* = 3, 8.8%), lighter fluids (*n* = 3, 8.8%), other types of hydrocarbons (*n* = 3, 8.8%), and 1 fatality (2.9%) each for: diesel fuels, turpentine, and unknown types of hydrocarbons. Males (OR 1.09; 95% CI 1.05–1.13) and children < 6 years old (OR 1.19; 95% CI 1.13–1.26) were more likely to experience a serious medical outcome than females and youth 6–19 years old, respectively.

### Hydrocarbon categories

Among youth < 20 years old, the gasolines category was the most common hydrocarbon category associated with ingestion cases (24.6%), followed by lubricating oils and/or motor oils (19.9%), other types of hydrocarbons (14.9%), lamp oils (11.3%), and lighter fluids and/or naphtha (10.3%) (Table 2). However, the relative ranking of hydrocarbon categories varied by age group; for example, lubricating oils and/or motor oils accounted for higher proportion of cases (22.1%) than gasolines (19.6%) among children < 6 years old. The proportion of cases attributable to gasolines increased with increasing age group, ranging from 19.6% among children < 6 years old to 58.3% among teenagers 13–19 years old. In contrast, the proportions of cases attributable to lubricating oils and/or motor oils, other types of hydrocarbons, and lamp oils decreased with increasing age group. The relative ranking of hydrocarbon categories among individuals < 20 years old also varied by reason for exposure. Suspected suicides were associated most often with gasolines (24.6%), followed by lighter fluids (17.0%) and lubricating oils (14.7%). The hydrocarbon categories most frequently associated with abuse were Freon and other propellants (54.7%), followed by gasolines (26.7%) and unknown types of hydrocarbons (4.0%).

**Table 2:** First-ranked hydrocarbon ingestions among individuals younger than 20 years old reported to the National Poison Data System by hydrocarbon category and age group, 2000–2021

Hydrocarbon categories	Age groups			
	< 6 years old *n* (%)*	6–12 years old *n* (%)*	13–19 years old *n* (%)*	Total *n* (%)*
Gasolines	46,158 (19.6)	5929 (34.5)	17,756 (58.3)	70,007 (24.6)
Lubricating oils and/or motor oils	52,132 (22.1)	2373 (13.8)	2089 (6.9)	56,632 (19.9)
Lamp oils	30,490 (12.9)	840 (4.9)	716 (2.3)	32,054 (11.3)
Lighter fluids and/or naphtha	26,087 (11.1)	1050 (6.1)	2040 (6.7)	29,204 (10.3)
Mineral spirits	12,185 (5.2)	877 (5.1)	1161 (3.8)	14,238 (5.0)
Kerosenes	11,807 (5.0)	726 (4.2)	680 (2.2)	13,219 (4.7)
Freon and other propellants	4645 (2.0)	2101 (12.2)	2149 (7.1)	8952 (3.2)
Diesel fuels	3076 (1.3)	308 (1.8)	854 (2.8)	4254 (1.5)
Turpentine	2245 (1.0)	200 (1.2)	345 (1.1)	2799 (1.0)
Toluene or xylene (excluding adhesives)	2220 (0.9)	200 (1.2)	244 (0.8)	2665 (0.9)
Mineral seal oil	775 (0.3)	20 (0.1)	20 (0.1)	816 (0.3)
Benzene	0 (0.0)	0 (0.0)	2 (0.0)	2 (0.0)
Other types of halogenated hydrocarbons	1200 (0.5)	94 (0.5)	119 (0.4)	1415 (0.5)
Other types of hydrocarbons	38,189 (16.2)	2156 (12.6)	2007 (6.6)	42,393 (14.9)
Unknown types of hydrocarbons	4848 (2.1)	292 (1.7)	288 (0.9)	5435 (1.9)

*Percentages may not add to 100.0% due to rounding error

Ingestion of lamp oils was associated with the greatest proportion of serious medical outcome (29.6%), followed by lighter fluids and/or naphtha (17.5%) and gasolines (15.0%). Similarly, the proportion of HCF admissions was highest for ingestions involving lamp oils (32.0%), followed by lighter fluids and/or naphtha (17.9%) and gasolines (12.5%). Compared with other substances, lamp oils were more likely to be associated with a serious medical outcome (OR 3.64; 95% CI 3.50–3.79), death (OR 4.32; 95% CI 2.13–8.73), or admission to a CCU or non-CCU (OR 4.38; 95% CI 4.21–4.57). In addition, lamp oils were more likely than other hydrocarbons to be associated with aspiration (with ingestion) (OR 3.14; 95% CI 2.99–3.30) than ingestion without aspiration.

### Trends

**Fig. 1:**
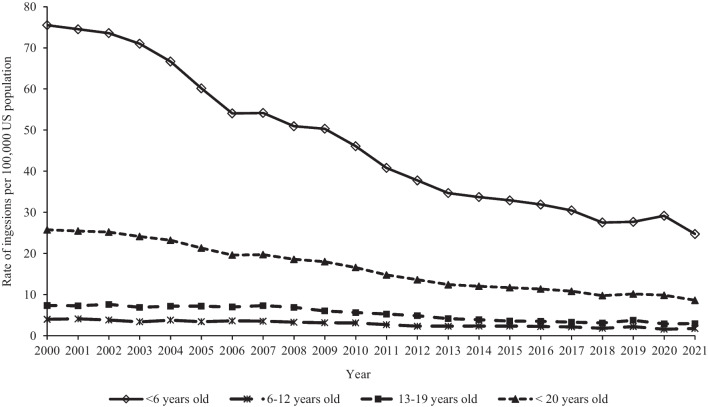
Annual rate of hydrocarbon ingestions reported to United States Poison Centers by age group, National Poison Data System 2000–2021

**Fig. 2:**
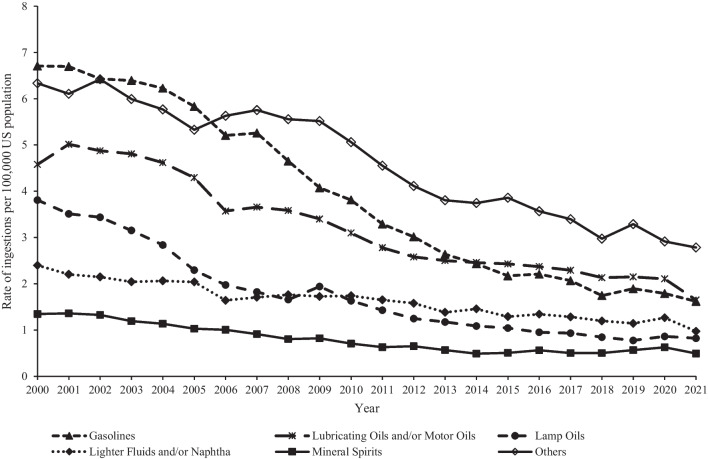
Annual rate of hydrocarbon ingestions reported to United States Poison Centers by substance category, National Poison Data System 2000–2021

**Fig. 3:**
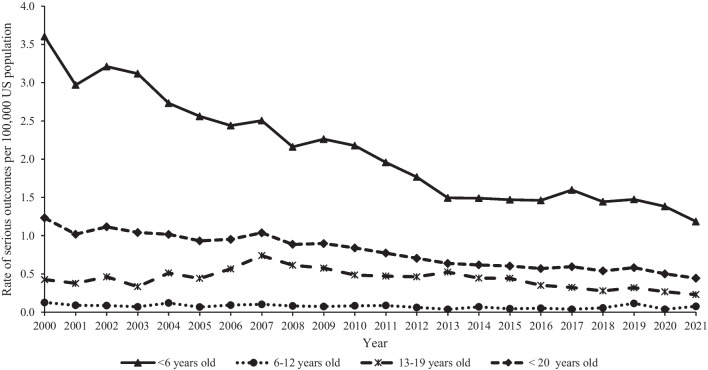
Annual rate of serious medical outcomes due to hydrocarbon ingestions reported to United States Poison Centers by age group, National Poison Data System 2000–2021

Trend analyses included all hydrocarbon ingestion cases (*n* = 297,640, including first-ranked and non-first-ranked cases) reported to US PCs from 2000 to 2021. The rate of hydrocarbon ingestions among youth < 20 years old per 100,000 US population decreased significantly by 66.5% (*p* < 0.0001) from 25.8 in 2000 to 8.6 in 2021 (Fig. 1). This trend was similar for males and females and all age groups, with the greatest decrease reported among males (− 67.7%, *p* < 0.0001) from 32.4 in 2000 to 10.5 in 2021 and children < 6 years old (− 67.2%, *p* < 0.0001) from 75.5 in 2000 to 24.8 in 2021 (trend by sex not shown in figure). The rate of ingestion decreased among all hydrocarbon categories, with the greatest decrease observed among lamp oils (− 78.4%, *p* < 0.0001) from 3.8 in 2000 to 0.8 in 2021, followed by gasolines (− 75.9%, *p* < 0.0001) and lubricating oils and/or motor oils (− 64.0%, *p* < 0.0001) (Fig. 2). The rates of a serious medical outcome and admission to a CCU or non-CCU decreased significantly by 63.9% (*p* < 0.0001) from 1.2 in 2000 to 0.4 in 2021 and 64.3% from 5.5 in 2000 to 2.0 in 2021, respectively. Among the age groups, children < 6 years old demonstrated the greatest decrease in the rate of a serious medical outcome (− 67.1%, *p* < 0.0001) from 3.6 in 2000 to 1.2 in 2021 and rate of admission to a CCU or non-CCU (− 65.7%) from 16.1 in 2000 to 5.5 in 2021 (Fig. 3).

## Discussion

During the 22-year study period, there were 284,085 hydrocarbon ingestion cases reported to US PCs involving an individual < 20 years old and in which a hydrocarbon was the first-ranked substance; this equals an average of 12,913 individuals per year or one person every 41 min. Most (83.2%) cases involved children < 6 years old, and consistent with this age group, most (89.8%) ingestions were related to exploratory behavior (an unintentional-general reason for exposure), involved a single substance (98.3%), and occurred in the home (96.5%). This information is consistent with previous findings and is helpful for targeting prevention efforts (Jolliff et al. 2013; Tenenbaum et al. 2021; Sheridan et al. 2021). Strategies for primary prevention of hydrocarbon ingestion among young children include (1) use of opaque, child-resistant containers at the point of sale, (2) avoidance of use of colors, scents, and container appearance that could lead to confusion with juices or other beverages, (3) do not transfer the product to another container, (4) secure the closure of the container after each use, (5) storage in a preferably locked location that is up, away, and out of sight and reach of young children, especially if stored in the home, which is where young children spend much of their time, (6) consider storing hydrocarbons outside the home, (7) do not use high-risk products, such as oil lamps, in homes where young children live or visit, and (8) appropriate adult supervision. (Forrester 2022; Jolliff et al. 2013; Schwebel and Swart 2009; Sheikh et al. 2013; Tenenbaum et al. 2021; Perrot and Palmer 1992; Murthy et al. 2018; Hoffman et al. 2004)

In 2001, the US Consumer Product Safety Commission (CPSC) promulgated a rule under the authority of the Poison Prevention Packaging Act (PPPA) (Law 1970) that mandated child-resistant packaging for liquid household products that contain low-viscosity hydrocarbons (United States Consumer Product Safety Commission 2001). This federal rule went into effect in October 2002 and likely contributed to the observed decrease in the rate of hydrocarbon ingestion among children < 6 years old by 67.2% from 2000 to 2021. However, the overall rate decrease was gradual during the study period with a slight acceleration in the decline from 2004 to 2006 (Fig. 1). This contrasts with the more robust response to the enactment of the PPPA of 1970. The PPPA, which required child-resistant closures for selected toxic products, resulted in a decrease in ingestion of baby aspirin and other regulated non-baby aspirin products, including household chemicals, by 40% to 55% (Clarke and Walton 1979; Walton 1982). This decrease in response to the PPPA was rapid, occurring during the first 2–3 years following changes in child-resistant closures (Clarke and Walton 1979). Our study’s data began in 2000, and therefore, we were unable to adequately determine the trend of the rate of hydrocarbon ingestion among young children prior to promulgation of the CPSC rule in 2001. Thus, although the CPSC rule likely contributed to the observed decline in hydrocarbon ingestions among young children, the magnitude of its influence is unclear. Another factor that may mitigate the effect of this regulation is that, although important, requiring child-resistant closures on hydrocarbon containers is only one step in the chain of protection needed to reduce hydrocarbon ingestions among children. The end product in which the hydrocarbon is used, such as an oil lamp, must also be child resistant and located out of a child’s reach.

Consistent with previous publications demonstrating the severe clinical outcomes of hydrocarbon aspiration (Ghezzi et al. 2019; Perrot and Palmer 1992), aspiration (with ingestion) in this study was approximately 16 times more likely to be associated with a serious medical outcome or admission to a CCU or non-CCU, and about 100 times more likely to be associated with death, than ingestion of a hydrocarbon without aspiration. In addition, lamp oils were three times more likely than other hydrocarbons to be associated with aspiration (with ingestion). Therefore, compared with other hydrocarbon categories, lamp oils were > 3 times more likely to be associated with a serious medical outcome, > 4 times more likely to be associated with admission to a CCU or non-CCU, and > 4 times more likely to be associated with death.

In 2011, the CPSC was petitioned with a request to require opaque packaging for lamp oil and torch fuel based on the concern that the appearance of these products could lead young children to mistake them for juice (United States Consumer Product Safety Commission 2011). Although torch fuel and lamp oil were already required to be sold in child-resistant packaging since 2002, this petition sought the additional requirement of opaque packaging, which is a strategy that has been employed for other products deemed attractive and dangerous to young children, such as cannabis edibles and liquid laundry detergent packets (Gaw et al. 2019; Onders et al. 2016; Valdez et al. 2014). In 2012, the CPSC voted to defer the petition to the development of a voluntary standard by ASTM International (United States Consumer Product Safety Commission 2012). That standard, F3304-22, was implemented in September 2021 and updated in August 2022 and specifies the design and performance requirements related to packaging, closures, and product labeling for “pourable lamp fuel and torch fuel containers with a rated capacity of less than 5 gallons intended for household use” (ASTM International 2022). There was a gradual decrease in the rate of ingestion of lamp oils from 2009 to 2019 (Fig. 2), but data from this study are not able to determine the influence of this voluntary standard on the ingestion of lamp oils among young children.

Our study agrees with the overall decreasing trend found by Forrester (Forrester 2022) for lamp oil injuries among children < 6 years old treated in US emergency departments between 2001 and 2019; however, that study reported an increase in cases from 2014 to 2017. Reasons that our findings do not reflect this increase include that (1) the study populations and surveillance methods are different (the National Electronic Injury Surveillance System is an active surveillance system involving a national probability sample of emergency departments and the NPDS is a passive surveillance system involving poison centers), and (2) the annual estimates reported in the Forrester study were small and potentially unstable, except in 2001.

Teenagers 13–19 years old accounted for 10.7% of cases. Although unintentional reasons for exposure also predominated in this age group similar to younger children, 17.8% were attributable to an intentional reason. Suspected suicide, misuse, and abuse were more common, which has also been previously described for other types of poisoning among teenagers (Becker et al. 2022; Chen et al. 2021). Impulsivity, risk-taking behavior, and other factors associated with immature executive functions and inexperience likely contribute to the higher proportion of intentional hydrocarbon ingestions in this age group (Ghezzi et al. 2019; Rajesh et al. 2014). The public health strategies for prevention of hydrocarbon ingestions among teenagers should take these developmental differences into consideration.

### Study limitations

This study has several limitations. The NPDS is a passive surveillance system, and therefore, underestimates the incidence of hydrocarbon ingestion nationally. Data are self-reported and cannot be completely verified by the poison centers or America’s Poison Centers, which could lead to misclassification and potential bias. Cases reported to the NPDS do not necessarily represent a poisoning or overdose. Repeat exposures involving the same individual were not identifiable because we did not have access to personal identifiers. Despite these limitations, the NPDS provides a comprehensive national database useful for investigating the characteristics and trends of hydrocarbon ingestions among individuals < 20 years old in the US.

## Conclusions

Although the rate of hydrocarbon ingestions decreased during the study period and most reported cases resulted in non-serious outcomes that required little to no health care involvement, the number of cases remains high with a non-trivial minority (4.5%) of cases associated with a serious medical outcome, including 34 deaths during the study period. Thirty-two of these deaths were among children < 6 years old and most were associated with aspiration (with ingestion). This underscores the need to increase primary prevention efforts, especially for young children.

## Data Availability

Data analyzed in this study were from the National Poison Data System, which is a proprietary database owned and managed by America’s Poison Centers. Data requests should be submitted to America’s Poison Centers at: https://www.aapcc.org/national-poison-data-system.

## Abbreviations

CPSC: United States Consumer Product Safety Commission
CCU: Critical Care Unit
CI: Confidence Interval
HCF: Health Care Facility
NPDS: National Poison Data System
OR: Odds Ratio
PC: Poison Center
RR: Risk Ratio
US: United States

## References

[CR1] American Association of Poison Control Centers. National Poison Data System Coding Users’ Manual. 2014; https://prod-knowledge-repository.s3-us-gov-west-1.amazonaws.com/references/NPDS%20Coding%20Users%20Manual%20%28May%202014%29.pdf?fbclid=IwAR0t4qYaQXGdV9HtOsDh-8IQt6y1dZ8bJGcocTi1t4AzfhD7mrU8YwOMtnA, 24 Apr 2023.

[CR4] America's Poison Centers. National Poison Data System. https://www.aapcc.org/national-poison-data-system. Accessed 20 Sept 2023.

[CR2] ASTM International. Standard specification for lamp fuel and torch fuel packaging, ASTM F3304-22. 2022; https://www.astm.org/f3304-22.html. 24 Apr 2023.

[CR3] Becker S, Spiller HA, Badeti J (2022). Cocaine exposures reported to United States poison control centers, 2000–2020. Clin Toxicol.

[CR5] Chen T, Spiller HA, Badeti J, Funk AR, Zhu M, Smith GA (2021). Methamphetamine exposures reported to United States poison control centers, 2000–2019. Clin Toxicol.

[CR6] Clarke A, Walton WW (1979). Effect of safety packaging on Aspirin ingestion by children. Pediatrics.

[CR7] Cox R, Amundson T, Brackin B (2008). Evaluation of the patterns of potentially toxic exposures in Mississippi following Hurricane Katrina. Clin Toxicol (phila).

[CR8] Forrester MB (2022). Pediatric lamp oil injuries treated in US Emergency Departments. Pediatr Emerg Care.

[CR9] Gaw CE, Spiller HA, Casavant MJ, Chounthirath T, Smith GA (2019). Safety interventions and liquid laundry detergent packet exposures. Pediatrics.

[CR10] Ghezzi M, Odoni M, Testagrossa O (2019). Pneumonia in a teenager hiding a fire-eating stunt. Pediatr Emerg Care.

[CR11] Gummin DD, Mowry JB, Beuhler MC (2021). 2020 Annual Report of the American Association of Poison Control Centers' National Poison Data System (NPDS): 38th Annual Report. Clin Toxicol (phila).

[CR12] Hoffman RJ, Morgenstern S, Hoffman RS, Nelson LS (2004). Extremely elevated relative risk of paraffin lamp oil exposures in orthodox Jewish children. Pediatrics.

[CR13] Jayashree M, Singhi S (2011). Changing trends and predictors of outcome in patients with acute poisoning admitted to the intensive care. J Trop Pediatr.

[CR14] Jolliff HA, Fletcher E, Roberts KJ, Baker SD, McKenzie LB (2013). Pediatric hydrocarbon-related injuries in the United States: 2000–2009. Pediatrics.

[CR16] Makrygianni EA, Palamidou F, Kaditis AG (2016). Respiratory complications following hydrocarbon aspiration in children. Pediatr Pulmonol.

[CR17] Murthy S, Das S, Hanuman BS (2018). Fatal diesel poisoning: A case report and brief review of literature. Am J Forensic Med Pathol.

[CR18] Onders B, Casavant MJ, Spiller HA, Chounthirath T, Smith GA (2016). Marijuana exposure among children younger than six years in the United States. Clin Pediatr.

[CR19] Perrot LJ, Palmer H (1992). Fatal hydrocarbon lipoid pneumonia and pneumonitis secondary to automatic transmission fluid ingestion. J Forensic Sci.

[CR15] Public Law 91-601 S, December 30, 1970, as amended. Poison Prevention Packaging Act. 1970. http://www.cpsc.gov/s3fs-public/pdfs/blk_media_pppa.pdf. 24 Apr 2023.

[CR20] Rajesh Z, Michael C, Ryan S (2014). Lungs ablaze: an unusual case of aspiration pneumonitis. Chest.

[CR21] Reddy MV, Ganesan SL, Narayanan K (2020). Liquid mosquito repellent ingestion in children. Indian J Pediatr.

[CR22] Schwebel DC, Swart D (2009). Preventing paraffin-related injury. J Inj Violence Res.

[CR23] Sheikh S, Chang A, Kieszak S (2013). Characterizing risk factors for pediatric lamp oil product exposures. Clin Toxicol (phila).

[CR24] Sheridan DC, Hughes A, Horowitz BZ (2021). Pediatric ingestions: new high-risk household hazards. Pediatr Rev.

[CR25] Slima S, Ragab E, Abdalgeleel S (2021). Evaluation of cases of kerosene poisoning: a 3-year prospective study at Menoufia University Hospitals. Ain-Shams J Forensic Med Clin Toxicol.

[CR26] Tenenbaum A, Rephaeli R, Cohen-Cymberknoh M, Aberbuch D, Rekhtman D (2021). Hydrocarbon intoxication in children: clinical and sociodemographic characteristics. Pediatr Emerg Care.

[CR27] United States Census Bureau. National intercensal datasets: 2000–2010. https://www.census.gov/data/datasets/time-series/demo/popest/intercensal-2000-2010-national.html. Accessed 20 Sept 2023.

[CR28] United States Census Bureau. National population by characteristics: 2010-2019. https://www.census.gov/data/tables/time-series/demo/popest/2010s-national-detail.html. Accessed 20 Sept 2023.

[CR29] United States Census Bureau. National population by characteristics: 2020–2022. https://www.census.gov/data/datasets/time-series/demo/popest/2020s-national-detail.html. Accessed 20 Sept 2023.

[CR30] United States Consumer Product Safety Commission. 16 CFR Part 1700. Petition requesting non-see-through packaging for torch fuel and lamp oil. federal register. 2011; 76(143): 44506–44507; https://www.govinfo.gov/content/pkg/FR-2011-07-26/pdf/2011-18512.pdf. 24 Apr 2023.

[CR31] United States Consumer Product Safety Commission. Household Products Containing Hydrocarbons; Final Rules. Federal Register. 2001 (01-26837): 53951–53957; https://www.govinfo.gov/content/pkg/FR-2001-10-25/pdf/01-26837.pdf, 24 Apr 2023.

[CR32] United States Consumer Product Safety Commission. Record of Commission Action on Petition PP11-1; Torch Fuel and Lamp Oil Petition. October 3, 2012. https://www.cpsc.gov/s3fs-public/RCA-Petition-PP-11-1-Torch-Fuel-and-Lamp-Oil-Petition-October-3-2012.pdf?VersionId=icZYzav9DtghzOGLYCGWeuDMqJBQKg_0. 12 May 2023.

[CR33] Valdez AL, Casavant MJ, Spiller HA, Chounthirath T, Xiang H, Smith GA (2014). Pediatric exposure to laundry detergent pods. Pediatrics.

[CR34] Walton WW (1982). An evaluation of the Poison Prevention Packaging Act. Pediatrics.

